# A closed loop two cycle audit investigating the availability and accessibility of physical healthcare equipment on forensic inpatient wards within mersey care's secure division

**DOI:** 10.1192/bjo.2021.236

**Published:** 2021-06-18

**Authors:** Thomas Davies, Stephen Owens, Tonia Forjoe

**Affiliations:** Merseycare NHS Foundation Trust

## Abstract

**Aims:**

To evaluate the provision of recommended medical equipment on forensic psychiatric inpatient wards in Mersey Care's secure division, as outlined by the Care Quality Commission (CQC) in their 2019 guidance “Brief Guide: Physical Healthcare In Mental Health Settings”. It has been documented that people with severe and enduring mental illness are at risk of dying on average 15 to 20 years earlier than people without, two thirds of which are due to avoidable physical illnesses. It was our aim to use these data to improve the provision of physical healthcare equipment on the wards of Mersey Care's secure division, in turn allowing for the safe assessment of patients in the acute setting, and the monitoring their chronic health conditions.

**Method:**

We conducted a closed loop, two cycle audit of all forensic inpatient wards in Mersey Care's secure division measuring the provision of physical health equipment against the CQC's 2019 guidance. The intervention was to present our findings and implement physical health equipment boxes in the clinic rooms on the wards. Low, medium, high, and secure learning disability (LD) wards were audited, with a control sample of non-secure wards (addiction, old age, general adult, and LD non-secure) in the initial cycle for comparison.

**Result:**

On initial audit, the mean availability of equipment across the secure division was 66% (range 50.9%-88.9%), and 75% across our sample of wards in the non-secure divisions (range 61.1%-88.9%). Following the intervention in the secure units, the mean availability increased to 73.5% (range 72.2%-77.8%). The mean percentage increase in equipment availability following intervention was 12.5% (range -12.5% to 41.8%).

**Conclusion:**

Following the intervention, the re-audit conducted found an overall improvement with 73.5% of recommended equipment available. Despite this improvement in equipment availability in the secure unit wards, the equipment is still less available than on the non-secure control wards. Due to this, further intervention and another re-audit have been planned. In the second cycle, significant items such as disposable gloves, pulse oximeters, sphygmomanometers, thermometers and stethoscopes were available across all wards. This was an improvement from the initial audit and allows for the safe assessment of patients in the acute setting.

